# Utilizing a church-based platform for mental health interventions: exploring the role of the clergy and the treatment preference of women with depression

**DOI:** 10.1017/gmh.2021.4

**Published:** 2021-02-19

**Authors:** Theddeus Iheanacho, Ujunwa Callista Nduanya, Samantha Slinkard, Amaka Grace Ogidi, Dina Patel, Ijeoma Uchenna Itanyi, Farooq Naeem, Donna Spiegelman, Echezona E. Ezeanolue

**Affiliations:** 1 Yale University, New Haven, CT, USA; 2 Center for Translation and Implementation Research, College of Medicine, University of Nigeria, Enugu Campus, Enugu, Nigeria; 3 Department of Psychiatry, University of Nigeria Teaching Hospital, Enugu, Nigeria; 4 Healthy Sunrise Foundation, Las Vegas, NV, USA; 5 Department of Community Medicine, University of Nigeria, Enugu Campus, Enugu, Nigeria; 6 University of Toronto, Toronto, Canada

**Keywords:** Church, clergy, global mental health, Nigeria, perinatal depression

## Abstract

**Background:**

Training lay people to deliver mental health interventions in the community can be an effective strategy to mitigate mental health manpower shortages in low- and middle-income countries. The healthy beginning initiative (HBI) is a congregation-based platform that uses this approach to train church-based lay health advisors to conduct mental health screening in community churches and link people to care. This paper explores the potential for a clergy-delivered therapy for mental disorders on the HBI platform and identifies the treatment preferences of women diagnosed with depression.

**Methods:**

We conducted focus group discussion and free-listing exercise with 13 catholic clergy in churches that participated in HBI in Enugu, Nigeria. These exercises, guided by the *positive, existential, or negative* (*PEN-3*) cultural model, explored their role in HBI, their beliefs about mental disorders, and their willingness to be trained to deliver therapy for mental disorders. We surveyed women diagnosed with depression in the same environment to understand their health-seeking behavior and treatment preferences. The development of the survey was guided by the *health belief model*.

**Results:**

The clergy valued their role in HBI, expressed understanding of the bio-psycho-socio-spiritual model of mental disorders, and were willing to be trained to provide therapy for depression. Majority of the women surveyed preferred to receive therapy from trained clergy (92.9%), followed by a psychiatrist (89.3%), and psychologist (85.7%).

**Conclusion:**

These findings support a potential clergy-focused, faith-informed adaptation of therapy for common mental disorders anchored in community churches to increase access to treatment in a resource-limited setting.

## Background

Mental disorders such as depression and anxiety are as prevalent in low and middle-income countries (LMICs) as they are in high-income, developed countries (Gureje *et al*., 2006; Bromet *et al*., 2011; Andrade *et al*., 2013). These affective disorders disproportionately affect women (Bebbington, 1998; Singleton *et al*., 2003) and significantly contribute to maternal morbidity, poor infant health, and lost economic opportunities (Schulz *et al*., 2000; Patel *et al*., 2004; Rahman *et al*., 2004; Adewuya *et al*., 2008; Tripathy *et al*., 2010).

In Nigeria, as in other sub-Saharan African countries, an estimated 10–20% of women experience depression during pregnancy and the postnatal period (Uwakwe, 2003; Adewuya *et al*., 2005; Abiodun, 2006). However, only 10% of adults with any mental health disorder in Nigeria receive any care irrespective of severity (Gureje and Lasebikan, 2006; Gureje *et al*., 2006). This is attributable in part to the severe lack of trained psychiatric specialists and the absence of supporting infrastructures for mental health care delivery (Saraceno *et al*., 2007; Organization, 2009). With only about 250 psychiatrists for a population of more than 180 million people, Nigeria exemplifies the severe lack of capacity for mental healthcare provision seen in LMICs (Kakuma *et al*., 2011; Nigeria, 2018).

Recently, increasing attention has focused on training non-psychiatric health workers and lay people to deliver mental health interventions with tools like the World Health Organization's (WHO) mental health Gap Action Plan-Intervention Guide (mhGAP-IG 2.0) (Organization, 2010; van Ginneken *et al*., 2013). The healthy beginning initiative (HBI) is an innovative platform that uses this approach. The HBI is a congregation-based initiative that utilizes volunteer church-based lay health advisors for health screening among pregnant women and their partners and linking them to care (Ezeanolue *et al*., 2013; Ezeanolue *et al*., 2015). Our team successfully integrated general mental health screening into the HBI framework (Iheanacho *et al*., 2015). The HBI platform has also been used to screen for malaria, sickle cell, and infant HIV infection (Gunn *et al*., 2015; Burnham-Marusich *et al*., 2016; Pharr *et al*., 2016). There is evidence that anchoring health interventions in community faith-based institutions is effective, especially in underserved communities and low-resource settings (DeHaven *et al*., 2004; Schoenthaler *et al*., 2018). Available data from Nigeria and other LMICs, although sparse, suggest that utilizing this approach with interventions for mental disorders and chronic diseases is a feasible, acceptable, and potentially sustainable pathway to closing the gap in access to effective treatments for these conditions in Nigeria (Abanilla *et al*., 2011; Ajayi *et al*., 2013).

Our study explores the potential for a clergy-led intervention for depression among women in southeastern Nigeria. This approach is based on the fact that a majority of adults in Nigeria are comfortable confiding in their clergy about emotional and mental health issues. An estimated 61% of adults in southeastern Nigeria attending a psychiatric clinic report they sought help from their clergy before seeing a mental health specialist (Odinka *et al*., 2014). Additionally, most clergy are already trained in pastoral counseling with some training on basic human psychology (Mucherera, 2006). Furthermore, clergy are well respected as thought leaders in the community (Conway, 2014). The catholic clergy who participated in HBI had a positive attitude about people experiencing mental illness. They also held the belief that depression and other mental disorders are treatable (Iheanacho *et al*., 2018).

This paper presents findings from (1) a qualitative study exploring the role of the clergy in HBI, their beliefs and attitudes about mental illness, and their willingness to be trained to deliver therapy for mental disorders with the supervision of psychiatric specialists on the HBI platform and (2) from a survey that sought to determine the treatment preferences of women diagnosed with depression and whether they would be willing to receive counseling/therapy from trained clergy.

## Method

We used a convergent, mixed-method approach that integrates qualitative and quantitative data from two different but convergent participant groups (Fetters *et al*., 2013).

### Free-listing exercise (FE) and focus group discussion (FGD) with clergy

For the qualitative data with clergy, we first used a written FE as a form of ‘mental inventory’ to identify the participants' cultural ‘salience’ on knowledge and beliefs about mental disorders (Liamputtong, 2019). In a freelist interview, a respondent simply lists members (things) that they perceive to be part of a domain (e.g. in our study question; what do you think are the causes of mental illness?) in whatever order they come to mind. The FE was followed by a FGD for further exploration of the clergy's role in HBI, their beliefs and attitudes about mental illness, and their willingness to be trained to deliver therapy for mental disorders with the supervision of psychiatric specialists.

The FE and FGD were guided by the positive, existential, or negative (PEN-3) cultural model.

#### Theoretical framework: the PEN-3 cultural model

The PEN-3 cultural model addresses the complexity of health issues by identifying cultural beliefs and practices that are critical to health behaviors, which should either be encouraged, acknowledged, and/or discouraged (Airhihenbuwa, 1995; Airhihenbuwa and Webster, 2004). The model consists of three dynamically interconnected and interdependent dimensions that contextualize the role of culture in shaping the understanding of and actions toward health and illness (Airhihenbuwa and Webster, 2004; Iwelunmor *et al*., 2014). These dimensions are *relationships and expectations*, *cultural empowerment*, and *cultural identity*. In this study, using a focus group setting, we explored the positive, existential, and negative aspects of the clergy's role in the HBI and their perception of mental illness in their communities. Although the positive aspects include values and relationships that promote health and a better knowledge of the mental illness, the existential examines the challenges of providing health services in churches, qualities of interactions with people with mental illness, and opportunities to be helpful. The negative aspects include health beliefs and actions that are harmful to those with mental illness and should be targeted for change. In utilizing the PEN-3 cultural model, this study identified clergy's positive beliefs and practices related to mental illness, existential interactions with those suffering from mental illness, and negative attitudes and stigmatizing behaviors that may serve as barriers to appropriate care for parishioners with mental disorders.

### Quantitative survey of women with depression

We used a 14-item questionnaire to identify beliefs about health-seeking for depression and treatment preferences among women diagnosed with depression. The development of the questionnaire was guided by the health belief model (HBM).

#### Theoretical framework: the health belief model

The HBM was developed in the early 50s to explain factors and variations mediating health behaviors (Hochbaum, 1958; Rosenstock, 1974). This model tries to explain why people decide for or against participation in public health programs, variations in treatment adherence, and has provided a guide to the design of health interventions that enhance compliance (Glanz *et al*., 2015). It integrates stimulus-response theory with cognitive theory in explaining behavior, thereby portraying that perception of reality rather than objective reality contributes largely to depicted behavior. It emphasizes the association of health behavior with personal characteristics and perceptions. The model consists of three interrelated components of perception, largely influenced by social and cultural context, which represents the various stages of the health decision process that influences health actions. These dimensions are perceived threat (perceived susceptibility and perceived seriousness), perceived benefit of the action, and perceived barriers to action (Glanz *et al*., 2015). In this study, using the HBM, we report the reactions of women to the presence of emotional disorder in the context of physical illness (perceived susceptibility); perception of treatment options, willingness to accept treatment from different care providers (perceived benefit of action); and the barriers from getting help (perceived barriers to action). The balance between these factors may suggest the women's likelihood of accepting the proposed intervention and their preferred course of action.

### Study setting and study population

The FGD and FE were conducted in Enugu, southeastern Nigeria in March 2015 with clergy from churches in the catholic diocese of Awgu. A total of 13 clergy participated in both exercises, which were held in the diocesan Bishop's court. All the clergy had participated in the HBI and spoke English and the local Igbo language fluently. Participants' demographics were captured using a sociodemographic questionnaire.

The quantitative survey was performed among women attending the Obstetrics and Gynecology clinics in the University of Nigeria Teaching hospital (UNTH), Ituku-Ozalla, Enugu State, Nigeria in April–May 2015. UNTH is the main tertiary hospital which serves the health care need of Enugu metropolis, including Awgu where HBI program is conducted. A total of 28 women with depression completed the survey. Participants' demographics were captured using a sociodemographic questionnaire.

The clergy and women did not receive any compensation for their participation in the FGD and survey completion.

### Data collection and study procedure

#### Qualitative data collection

The FGD was conducted in two sessions each lasting about 30 min. The first session focused on their participation in the HBI and the second session focused on their perceptions and beliefs about mental illness in Nigeria, their interactions with those affected in their communities, and their willingness to be trained to deliver therapy for mental disorders. Prior to data collection, and with input from HBI staff who worked with clergy, the research team developed the key initial research questions based on the relevant domains of the PEN-3 model. For the FGD, we explored the clergy's roles, experience, and thoughts related to HBI. Questions were framed using relevant topic guides to assess the benefits and impact of HBI to the community; enablers and barriers to implementation of HBI; and ways of sustaining the program. We further explored the perceptions participants may have had toward mental illness, the positive, existential, and negative factors that may influence them to have those perceptions, and the role of their interactions with their parishioners as well as the church, health systems, family, and community in influencing these perceptions. Probes were used in the focus groups as required. For the FE, after completing basic demographic data, participants were asked to respond to the following question: ‘What do you think of mental illness in Nigeria?’ Participants were asked to respond to the question with complete statements that they feel are important and relevant to the question. They were also asked to express their unique perspectives about the question using clear and concise statements that reflect their understanding of the subject. Interviewers used probes as necessary by asking for ‘anything more’ until the exercise was finished (see online Supplementary Appendix). Focus groups and FEs were conducted in English, although participants were encouraged to use the local Igbo language when they felt like it would help them express themselves better. These were then transcribed and translated into English for data analysis.

#### Quantitative data collection

The respondents were women attending the Obstetrics and Gynecology clinics in University of Nigeria Teaching Hospital Ituku-Ozalla Enugu diagnosed with depression following usual screening and evaluation procedures in the clinic. Following the confirmation for the diagnosis of depression, research staff will meet with the women to explain the research study, obtain informed consent, and give them the research questionnaire.

This was a pen and paper survey worded in basic English with the option for verbal interpretation to the local Igbo language by research staff who are fluent in both languages (although no participant requested interpretation). It was self-completed by the respondents in private but research staff were available to answer questions. Completed forms were collected on the same day. Respondents had to have a clinical diagnosis of depression, be aged 18 years and above, with the ability to give informed consent.

### Data analysis

For the qualitative data; first, a raw transcript was produced from the FGD videos, then a clean transcript was produced following independent reviews of the videos by three of the authors, two of whom did not participate in the FGD. Transcripts, field notes, and statements from the FE were then collated. Content analysis was used to identify themes with categories defined by the PEN-3 theoretical model. Initial coding was performed by two members of the research team independently and corroborated by a third. The research team compared the coded data to ensure consistency. Two dimensions of the PEN-3 model were used to organize the coded data into the following categories: (1) relationships and expectations: perceptions, enablers, and nurturers; and (2) cultural empowerment: positive, existential, and negative.

For the quantitative survey, data were analyzed using the Statistical Package for Social Science version 21 (SPSS) after sorting and coding the questionnaire. Data obtained with the sociodemographic questionnaire and the survey questionnaire were subjected to descriptive statistical analysis. Results were displayed in frequencies and tables as appropriate.

### Ethical approval

The study was approved by the Nigerian National Health Research Ethics Committee and the Health and Research Ethics Committee (HREC) of the University of Nigeria Teaching Hospital Ituku-Ozalla, Enugu.

## Results

### FGD and FE with clergy

The FGD explored the perceptions and experiences of the 13 clergy members involved. The clergy were all male with an average age of 46 years and an average time in the priesthood of 14 years. Each of the clergy held undergraduate degrees in philosophy and theology, and all practice pastoral counseling in their parishes. Table 1 describes how the clergy's roles in HBI and beliefs about mental illness align with the PEN-3 model core components of relationships and expectations; and cultural empowerment.

**Table 1.** Themes from the FGD with clergy based on the PEN-3 theoretical model

Theme	Relationships/expectations	Cultural empowerment
Clergy role in HBI	*Perception(s)*: Clergy play a positive role in HBI in their parishes. HBI is valuable for improving health and reducing stigma. *Enabler(s)*: Clergy are leaders of HBI events at their parishes. Clergy provide health education to members. Celebration of mothers, family and pregnancy. Sensitization of members to participate in program. *Nurturer(s)*: Clergy feel they can bring specialists in to help HBI participants. Clergy feel the men(spouses) are more involved in the pregnancy journey Clergy provide spiritual support to pregnant women.	*Positive*: Clergy are respected leaders in their communities. The church is perceived as a place to provide health interventions *Existential*: Clergy can make announcements to and provide prayer for their parishioners *Negative*: Stigma toward ‘immoral’ behaviors may be held by individual Clergy Participation by other non-church members maybe hindered by distance.
Causes of mental illness	*Perception(s)*: Clergy show knowledge of biological causes of mental illness. Also show knowledge of folk beliefs about mental disorders among their parishioners. Clergy have positive concerns and attitude for patients with mental illness. *Enabler(s)*: Clergy already provide counseling, psychoeducation and make referral to psychiatric centers. *Nurturer(s)*: Clergy provide social support, check in on parishioners with emotional struggles.	*Positive:* Clergy understand that mental illness can have external (environmental) causes and internal (biological) causes. *Clergy understand predisposing and precipitating factors of mental illness.* *Existential:* Clergy require training on causes of different mental illnesses. *Negative*: Mental illness is believed to have supernatural causes.
Manifestation of mental illness	*Perception(s)*: Clergy’ knowledge of manifestation of mental illness is largely that of psychotic presentation They also understand the role of stress and trauma. *Enabler(s)*: Clergy have experience with individuals with mental illness. They can identify those who need help. *Nurturer(s)*: Clergy provide education on causes and manifestations and myths about mental illness.	*Positive*: Clergy are able to work closely with and observe parishioners for changes in behavior, thoughts, and emotions. *Existential:* Clergy require training on manifestations/symptoms of mental illnesses. *Negative:* Clergy believe mentally ill people are violent.
Treatment of mental illness	*Perception(s)*: Clergy believe that something can be done about mental illness (different interventions illustrated). *Enabler(s)*: Clergy can refer parishioners to psychiatric treatment. *Nurturer(s)*: Clergy attempt to follow-up with individuals they help with mental illness. Clergy are willing to be trained to provide evidence-based counseling.	*Positive:* Clergy believe counseling and treatment can be effective. *Existential:* Clergy already counsel people who hold folk, mythical beliefs. Clergy believe that mental illness can be treated but not cured. *Negative:* Perceived, inadequate psychiatric facilities in the state. Government seems unconcerned for people with mental illness.

### Clergy's roles in healthy beginning initiative

#### Perceptions

The clergy saw themselves as being integral to the HBI program and its successful implementation in the community. One participant stated, ‘we began with an announcement to the parish, involved in praying for the pregnant women at the baby shower, baby reception’. Another stated, ‘I was assisting in spreading the information, in publicity and encouraging the women’. Indicating their involvement in the planning phase, another participant stated, ‘I was there starting the program till the end… including the announcement, the selection of the coordinators…’. The clergy perceived HBI as valuable in alleviating the fears families in their parishes have about pregnancy-related health problems and reducing stigma toward testing for conditions like HIV and sickle cell disease. One participant stated, ‘I have also discovered that it is not only testing for HIV since there are other diseases that we test for (sickle cell disease, hepatitis) things we need to test for, so the stigma has been drastically reduced’.

#### Enablers

The clergy identified their role as spiritual counselors as helpful in the success of HBI in their church and community. One participant stated, ‘We actually encountered a situation where some people didn't want to come out because they were afraid that if they came out, people could hurt them, you know, spiritually, so I had to counsel them and make them understand that nothing of that sort could happen’. Another stated, ‘…they come from the nearest parishes to do the baby shower and they ask for me to pray for them and stay with them…’. Referring to the unifying role of a joint spiritual activity as an important part of HBI, one participant stated, ‘…in my parish, once a month we do that, we gather them after Mass, pray for them, celebrate them, and wait for the baby shower’.

#### Nurturers

The clergy described their roles in supporting the HBI program as a church-based health intervention. One stated, ‘So my job is to be there to assistant them. And when the family asks me to pray, I pray for them because you are an assistant you assisted them. But “no one knows all”, we don't know all… so we call physicians or health personnel to help, to talk to them…’.

### Clergy's beliefs about mental illness and interest in therapy training

#### Perceptions

The clergy described positive and potentially negative perceptions of the causes, manifestations, and treatment of mental illness. They offered biologically based causes and manifestations (positive perceptions), which may be addressed by behavioral therapy. Causes mentioned included stress, interpersonal abuse (sexual abuse), genetics, and alcohol and drug use. One participant shared an example of stress as a cause, ‘emotional breakdown… somebody lost many Naira that doesn't belong to him and he kept on thinking on how to repay… he couldn't contain it…’. The manifestations stated by the clergy included ‘erratic speech and laughter’, ‘hallucinations’, ‘abnormal phobias’, ‘sleepless nights’, and ‘poor reasoning’. The clergy shared culture-based beliefs about causes of mental illness (potentially negative perceptions), which may require specialized, culturally sensitive training to address. One participant stated that ‘mental illness can be inflicted through charms… I hear people talk about it in areas where we have witch doctors…’. Another mentioned that *ajo nmuo*, or evil spirits, are believed to cause mental illness. The clergy had positive perceptions of the types of treatments people may receive or try for their mental illness, including counseling, reduction of stress, healthier lifestyles, and ‘psychiatric attention in the hospital…’. However, a consensus was not reached as to whether the clergy believed mental illness can be ‘fully cured’. One participant stated, ‘(mental illness is) treatable, I think yes. It can be managed… it could involve some medication…’.

#### Enablers

The clergy described their experiences with people suffering from mental illnesses. They provide counseling for their parishioners, and one participant noted, ‘I have had to write a recommendation to a psychiatric hospital…I explain that I have given advice to this person and that advice is not working…’. Referrals to a psychiatric facility were uncommon, with some stating that these referrals occur two times every 2 to 3 years. The also addressed providing counseling to individuals who believe in folk magic (charms and evil spirits), with one participant stating, ‘I try to find the basis of their fear…Like I try to make them understand that people can't harm them from such distance the way they think’.

#### Nurturers

The clergy are supportive of individuals who come to them for counseling for mental health needs and attempt to follow-up on those they helped. One participant noted that ‘One experience I had, I spoke with somebody, had a session with her…She seemed to be coming nearer the church then, but there wasn't a follow-up option and she disappeared again’. When asked how they felt about individuals suffering from mental illness, they stated that they felt ‘pity’ for them, felt ‘helpless’ for them, or ‘afraid’ of them. A participant described it as, ‘…it depends on the kind (of mental illness) the person is suffering from…because there is a kind (of mental illness) the person will be suffering from and there will be chaos in the parish…the person can distract everything in the parish house, so it depends on the level’. The participants unanimously responded ‘yes’ when asked if they were interested in formal training to provide counseling for mental disorders. On further probing, they stated their interest in being mental health ‘first responders’ or ‘first helpers’ providing initial counseling and connecting those who need more help to psychiatric specialists.

### Survey of women with depression

A total of 28 ethnically Ibo women completed the study questionnaires with no missing values identified. The mean age of the women was 42 with ages ranging from 19 to 65 years. The majority of the women were educated past secondary education (67.9%) and half the women were employed (full-time: 42.9%; part-time: 7.1%) and half were unemployed (50%). The majority of the women were married (64.3%) and had at least one child (64.1%). Most women lived in urban areas (64.3%). All were recently diagnosed with depression and receiving psychiatric treatment at the time of the survey.

#### Perceived susceptibility

All the respondents acknowledged their struggle with depressive symptoms, were engaged in care, and were willing to consider other additional treatments or supports.

#### Perceived benefit of action

Overall, the women reported that their emotional problems could be treated by the various methods provided (medication, individual/group counseling, support from friends/family, and faith). All women reported that their emotional problems may be treated through faith. Additionally, a majority of the women reported a stronger preference for receiving mental health treatment from a trained clergy (92.9%), followed by a psychiatrist (89.3%) or psychologist (85.7%).

#### Perceived barriers to action

For a majority of the women, embarrassment (85.7%), fear of others' opinions (85.7%), or family disapproval (92.9%) were not barriers to them seeking care for their mental health concerns (Table 2).

**Table 2.** Treatment preferences and perceived barriers among women with depression

Question	Response	*N*	Percent (%)
Do you think your emotional problems could be helped with any of the following?	Medication		
	Yes	27	96.4
	I don't know	1	3.6
	Individual counseling		
	Yes	27	96.4
	No	1	3.6
	Group counseling		
	Yes	26	92.9
	No	2	9.1
	Family and friends support		
	Yes	27	96.4
	No	1	3.6
	Faith		
	Yes	28	100.0
Would you be willing to receive treatment including counseling for this emotional problem from any of the following?	General medical doctor		
	Yes	22	78.6
	No	6	21.4
	A general nurse		
	Yes	18	64.3
	No	10	35.7
	A psychologist		
	Yes	24	85.7
	No	4	10.7
	A psychiatrist		
	Yes	25	89.3
	No	3	10.7
	A chemist (patent medicine dealer)		
	Yes	5	17.9
	No	23	82.1
	A faith leader trained for counseling		
	Yes	26	92.9
	No	2	7.1
	A traditional healer		
	Yes	3	10.7
	No	25	89.3
Would any of the following keep you from getting help?	Being embarrassed to talk about personal matters with others		
	Yes	4	14.3
	No	24	85.7
	Being afraid of what others might think		
	Yes	5	17.9
	No	23	82.1
	Family members might not approve		
	Yes	2	7.1
	No	26	92.9

### Merged results

The qualitative data from the FGD with clergy showed their roles as counselors and their support for those experiencing stress and mental disorders within their communities. The data also showed they were willing to receive additional training to enhance their capacity to provide specific therapy for mental disorders. The quantitative data from the survey of women with depression in the same communities showed overwhelming preference for clergy-delivered interventions for mental disorders. Thus, there seemed to be a concordance in data suggesting that a clergy delivered intervention for a mental disorder like depression is acceptable in the community.

## Discussion

The results from this study show that clergy who participated in the HBI, a faith-based platform for community health interventions, had a positive view of the experience. They embraced their roles as facilitators and connectors between the program staff and their parishioners. They also identified the value of HBI in enhancing the health of pregnant women in their communities while supporting the work they do as clergy. This is an indication of the unique role of the church in this community; serving as a platform for early identification of illness through screening, brief health intervention, referral to higher levels of care, and community follow-up (Gunn *et al*., 2015; Pharr *et al*., 2016). This is particularly relevant for chronic diseases among underserved populations in resource-limited settings such as Nigeria. Extending the reach of the mental health professionals in the community by providing training and consultations to persons occupying a strategic role in that community like the clergy, has been an important element of the community mental health movement from its beginnings in closing treatment gap (Bissonette, 1979).

The clergy participants expressed perceptions of mental disorders that reflect an understanding of the biological and stress model of causation as well as the cultural context of mental illness in their communities. They have embraced their roles as counselors for those experiencing emotional difficulties sometimes providing psychoeducation to their families and connecting them to care. Furthermore, they were unanimously open to the idea of receiving training to specifically provide specific therapies and counseling for mental disorders on a faith-based platform like HBI. Thus, the potential for developing and testing a congregation-based, clergy-led therapy intervention anchored in the HBI community platform and connected to primary care and psychiatric specialists is promising. Additionally, they can leverage their positions as trusted leaders and confidants in their local communities in building therapeutic alliance, a key element in psychotherapies (Krupnick *et al*., 2006).

They also seemed to endorse some culturally accepted models of the causation of mental disorders that ascribed causation to supernatural forces including ‘evil spirits’, ‘charms’ and ‘witchcraft’. This dual (biological and supernatural), but contradictory perception of causation of mental illness has been noted in published literature and our previous studies on attitudes to mental disorders among different segments of the populace in Nigeria (Adewuya and Oguntade, 2007; Iheanacho *et al*., 2014; Iheanacho *et al*., 2016). This could be considered a potential challenge in developing or adapting orthodox therapy training tools based on the commonly accepted constructs of causation of mental disorders. However, findings from our prior study suggest that this may not be a hindrance as the clergy overwhelmingly believe that mental disorders are treatable (Iheanacho *et al*., 2018). Moreover, the ‘bio-psycho-socio-spiritual’ model has been used by many faith-based cultures and has informed successful adaptation of specific therapies (Naeem *et al*., 2019). The development of specific therapy training tools for the clergy in this context should include culturally relevant approaches that acknowledge this dual understanding of causation but still promote the core tenets of effective therapy (Naeem *et al*., 2019).

Results from our survey of women with perinatal depression in the same geographical area of Enugu showed that a vast majority would be open to receiving counseling or therapy from trained clergy. This is not surprising as most studies of pathways to mental health care in Nigeria show that consultation with the clergy prior to seeking formal care is common (Adeosun *et al*., 2013; Odinka *et al*., 2014). Furthermore, all the women ranked ‘faith’ as well as ‘individual counseling’ as important in treating their depression.

Taken together, these findings support the idea of exploring the feasibility of a clergy-led intervention for common mental disorders like depression using a well-tested, church-based platform like the HBI program. Our findings also add to existing literature from Benin City, Nigeria which shows that a majority of clergy from both Christian and Muslim backgrounds correctly identified mental disorders depicted in vignettes, embraced a multifactorial model of disease causation, and expressed willingness to collaborate with mental health care workers to deliver care (James *et al*., 2014). Furthermore, there is evidence that clergy-led interventions for mental disorders anchored in community faith-based organizations are feasible and acceptable approaches to increasing access to care in low resource settings (Hankerson *et al*., 2018). This community-based, task-shifting approach of interventions delivered by trained, lay health persons has been shown to be effective in low-income countries in Africa (Chibanda *et al*., 2016; Mutamba *et al*., 2018).

However, it is important to identify, understand, and work through the potential challenges that could arise as a result of this approach. These include moral and identity frictions at the intersection of faith, beliefs, culture, and human rights in developing and implementing clergy-led, faith-based interventions for mental disorders in collaboration with formal health care systems (Bissonette, 1979; Arias *et al*., 2016; Read, 2019).

One limitation of our study is worth noting. Although the development of the 14-item questionnaire completed by the women was guided by the well-established health behavior model with the results reflecting cultural norms in Nigeria (Lasebikan, 2016), its psychometric characteristics have not been fully tested. We plan to build on this pilot study by further exploring the questionnaire's factor structure and validating it within our study population in southeastern Nigeria.

## Conclusion

Clergy from the Awgu Catholic archdiocese who participated in the HBI, a platform that uses community churches for health interventions and linkage to care, had positive perceptions of their roles in the initiative and were willing to receive training to provide counseling for mental disorders on the HBI platform. Women diagnosed with depression in the same archdiocese overwhelmingly preferred to receive counseling from trained clergy. These findings support a potential clergy-focused, faith-informed adaptation of psychotherapies for common mental disorders, such as depression anchored in community churches to increase access to treatment in a resource-limited setting.

## Supplementary material

For supplementary material accompanying this paper visit http://dx.doi.org/10.1017/gmh.2021.4. click here to view supplementary material
