# Supplementary material for: Utilizing a church-based platform for mental health interventions: exploring the role of the clergy and the treatment preference of women with depression
Source: Glob Ment Health (Camb). 2021 Feb 19;8:e5. doi: 10.1017/gmh.2021.4 (PMC8127631; doi:10.1017/gmh.2021.4)
Supplement: Supplementary file 1 [file S2054425121000042sup001.docx]

**Focus Group Discussion questions and probes**

**Clergy involvement in the Church-Based Health programme, The Healthy Beginning Initiative(HBI)**

How are you involved in implementing Healthy Beginning Initiative?

What is the impact of the Healthy Beginning Initiative on the health setting of this community?

Do you think HBI has helped to reduce stigma?

Any additional points on impact of HBI?

What are the enablers and the barriers? Things that have made the implementation of HBI possible and what are the barriers you have?

What are the practical challenges or barriers that you have had?

How has HBI changed how you do things in the church? Has this really disrupted… Has it changed… has it caused you to move around things a little bit to accommodate it?

So, the general consensus is that the Healthy Beginning Initiative doesn’t entail a lot of serious changes to the way you do these things. So how about costs? Has HBI cost you financially or any other kinds of costs?

What are some benefits that you have seen?

How could we improve the Healthy Beginning Initiative?

One way could be because you know the Healthy Beginning Initiative is church-based, and we are talking about how to integrate the health center within that. Could it be… what format might that take?

Are there things that are outside of your control that may hinder the successful implementation of HBI?

Are there any additional constraints, challenges?

Any general thoughts on Healthy Beginning Initiative that we haven’t covered so far?

Anything in general pertaining to the Healthy Beginning Initiative?

**Clergy beliefs, understanding and role in interventions for mental disorders**

What is mental illness?

How does that present? What are the manifestations of mental illness?

What do you think are the causes of mental illness?

From your point of view, how does a charm cause mental illness?

And how does alcohol cause mental illness?

How do you explain that?

Someone mentioned stress, what do you think is the connection of mental illness and stress.

What do you think is the connection between the drug they use and the mental illness?

What treatments do you know that help with mental illness?

Some of you mentioned depression, anxiety, schizophrenia, even post-natal blues/depression. What are the things you know that can help? What are the treatments that are available?

So, based off of that, are mental illnesses treatable? Is depression treatable, is anxiety treatable? Is schizophrenia treatable? Is drug use disorder treatable?

Has any one of you ever met anyone who has mental illness, who suffers from any type of mental illness?

What do you see are common emotional health issues in the course of your work… working in the parish and seeing your parishioners, what is the commonest thing you think is emotional distress that you see?

When you come across your parishioners with mental health issues what do you do for them?

So, you provide counseling and support, is that something you all do, would do?

Now, how do you think the people that you have seen, that you have sent to people, that you have tried to get help, how are they doing? Overall? Did they get help, did they get a little better? Did they feel comforted? What was the outcome? What happened?

When you see someone or people who are suffering with mental illness, how do you feel? How does that make you feel?

If you had somebody with emotional problems or mental illness, or something going on with them, would you live with them?

If he or she is getting help and taking medication, would you live with him/her?

Would you be willing to be trained to provide counseling for mental disorders in your parishes?

What kind of training do you think you will need?

What role can you play as a clergy in the care of those with mental disorders
